# Impact of Pronator Quadratus Muscle Tear in Distal Radius Fractures on Patient Outcomes: Analyses of 55 Patients in a Volar Locking Plate Arm of a Randomized Clinical Trial

**DOI:** 10.7759/cureus.58576

**Published:** 2024-04-19

**Authors:** Morten Eikrem, Tom Lian, Jan Erik Madsen, Wender Figved

**Affiliations:** 1 Orthopaedic Department, Aalesund Hospital, Moere and Romsdal Hospital Trust, Aalesund, NOR; 2 Institute of Clinical Medicine, University of Oslo, Oslo, NOR; 3 Orthopaedic Department, Baerum Hospital, Vestre Viken Hospital Trust, Gjettum, NOR; 4 Division of Orthopaedic Surgery, Oslo University Hospital, Oslo, NOR

**Keywords:** surgical management of distal radius fractures, hand grip strength, osteosynthesis, pronator quadratus, distal radius fractures

## Abstract

Background

The advantage of pronator quadratus (PQ) repair following internal fixation via the volar approach in distal radius fracture (DRF) surgery remains unconfirmed in the literature. The aim of this study was to compare grip strength, patient-reported outcomes, and functional results between patients with an intact PQ and those with a ruptured PQ before undergoing surgery with a volar locking plate for dorsally displaced unstable extra-articular DRFs.

Methods

A total of 120 patients aged 55 years and older were included in a randomized controlled trial comparing a volar locking plate with a dorsal nail plate. Of the 60 patients randomized to the volar plate group, the integrity of the PQ muscle was recorded during surgery for 55 patients, who were included in this study. The outcomes measured were the Quick Disabilities of the Arm, Shoulder, and Hand Outcome Measure (QuickDASH) score, the Patient-Rated Wrist Evaluation (PRWE) score, the EQ-5D index, the visual analog scale (VAS) score, grip strength, and range of motion (ROM).

Results

The median age was 67 years (range 55 to 88), and the one-year follow-up rate was 98%. Patients with an identified intact PQ (28/55) before surgical release had better QuickDASH scores after one year (2.5 vs 8.0, mean difference 5.5, 95% CI: 1.3 to 9.8, p=0.028). Patients in the intact group also had better EQ-5D Index scores after one year (0.94 vs 0.85, mean difference 0.089, 95% CI: 0.004 to 0.174, p=0.031), and demonstrated better grip strength throughout the trial; after one year: 24 kg vs 20 kg (mean difference 3.9; 95% CI: 0.3 to 7.6, p=0.016). After one year, the intact group had regained 96% of their grip strength and the nonintact group had regained 93% of their grip strength compared to the uninjured side. The observed differences may be of questionable clinical importance, as they were lower than those of previously proposed minimal clinically important differences (MCIDs).

Conclusions

Patients with a DRF and a ruptured PQ prior to surgery exhibited higher QuickDASH scores and lower EQ-5D index scores after one year. The integrity of the PQ should be reported in future studies.

## Introduction

The volar approach has gained popularity after the introduction of low-profile locking plates for distal radius fractures (DRFs). This approach involves detaching the superficial head of the pronator quadratus (PQ) muscle from the radial and distal edge of the radius. Anatomic PQ repair after volar plating is disputed. A recent meta-analysis of randomized controlled trials (RCTs) comparing PQ repair versus no repair after volar plating of DRFs revealed no differences in functional outcome, range of motion (ROM), grip strength, or reoperation rate [1]. Fang et al. evaluated the healing of the PQ by direct observation intraoperatively during plate removal one year after the healing of the fracture in 126 patients [2]. Muscle healing was observed in 23 patients, but the muscles were significantly atrophic with scar hyperplasia and fibrosis. In one hundred three patients, PQ muscle fibers were not observed and were considered not healed. Nevertheless, healing of the muscle did not affect the Patient-Rated Wrist Evaluation (PRWE) score, grip strength, ROM, or isokinetic forearm rotation strength between the two groups.

The literature on the clinical relevance of PQ integrity prior to DRF stabilization with a volar plate, regardless of PQ repair, is scarce. We aimed to evaluate whether PQ integrity prior to surgery influences clinical and functional outcomes in patients who underwent DRF fixation via a volar approach. Our hypothesis was that PQ rupture after trauma prior to surgery does not influence outcomes.

## Materials and methods

**Figure 1:**
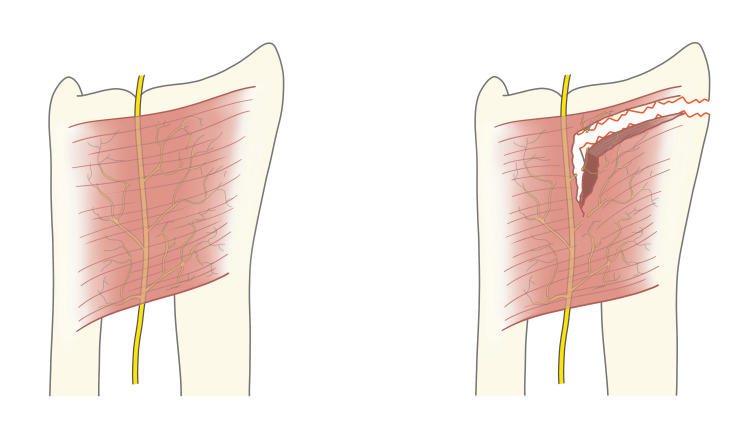
Pronator quadratus muscle Illustration showing intact (left) and ruptured (right) pronator quadratus muscle, with the anterior interosseous nerve of the forearm, a branch of the median nerve. Courtesy of A.I. Hellevik, MD, PhD, Aalesund, Norway.

The study population comprised one arm of a single-center RCT. This study was conducted at Baerum Hospital, Vestre Viken Hospital Trust, Norway, a Level-II trauma hospital with a catchment area population of 190,000 (2011), and a dorsal plate was compared with a volar plate for unstable extraarticular DRFs in patients aged 65 years and older [3]. In this RCT, we recorded the integrity of the PQ muscle in the volar plate group intraoperatively (Figure 1).

Ethics

The study protocol was designed according to the recommendations of the CONSORT Initiative [4] and was approved by the Regional Ethics Committee of Eastern Norway (ref. S-0862b) and the local data protectorate (ref. 09-2008SAB). The trial was registered at ClinicalTrials.gov (NCT00848263). This article was previously posted to the Research Square preprint server on March 18, 2024.

Enrollment

**Figure 2:**
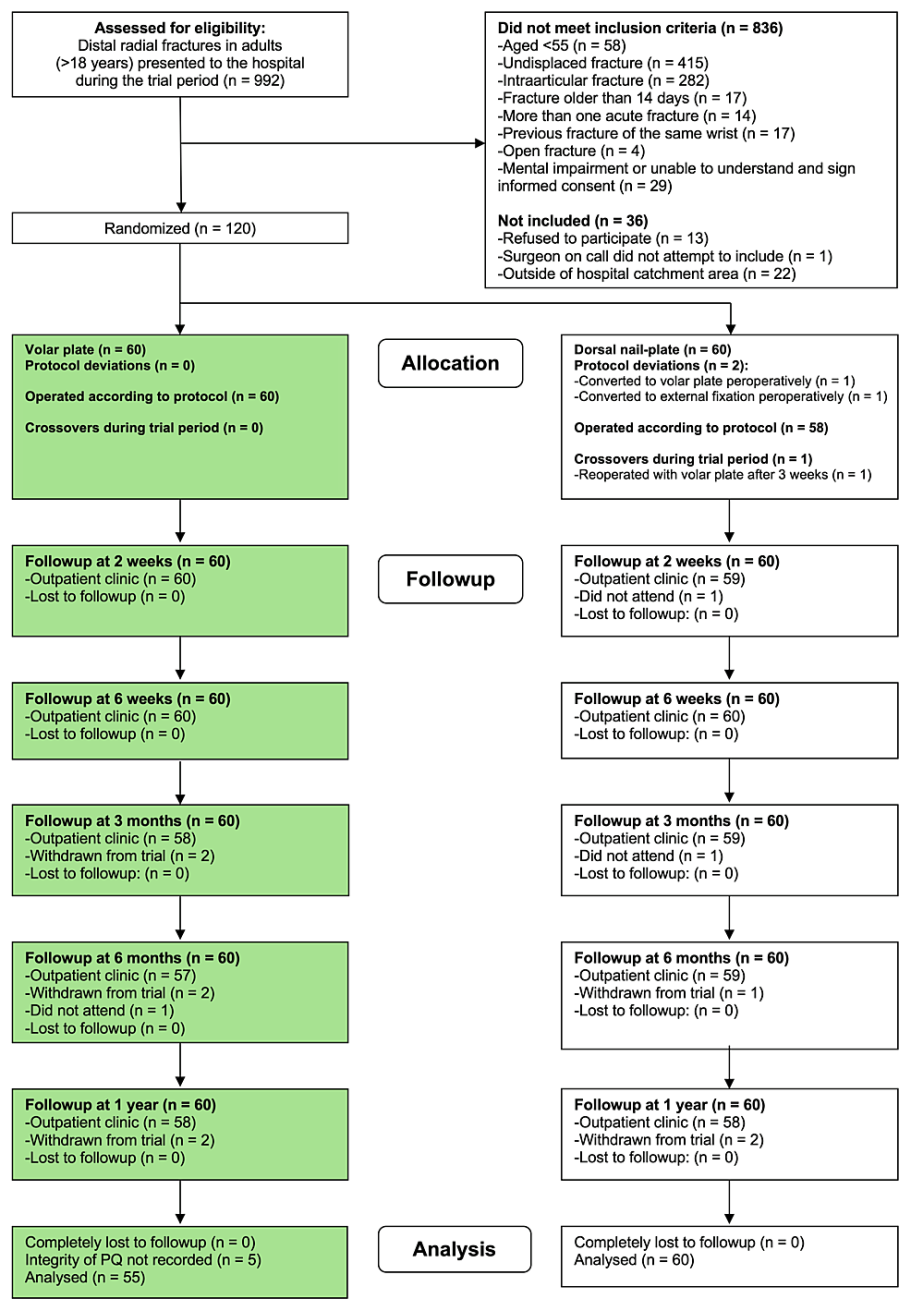
CONSORT flow diagram CONSORT (Consolidated Standards of Reporting Trials) flow diagram of the recruitment and flow of patients with distal radial fractures through the study. The patients included in this substudy are indicated in green boxes.

We evaluated patients aged 55 years and older with an unstable dorsally displaced fracture of the distal radius without articular involvement for enrollment. Instability was diagnosed after Lafontaine’s criteria [5], or observed redisplacement following adequate closed reduction. The exclusion criteria included a previous fracture of the same wrist, more than one acute fracture (except for the ulnar styloid process), an open fracture, a fracture older than 14 days, and mental impairment or inability to understand and sign an informed consent. A total of 120 patients were included between April 2009 and December 2012. The study arm randomized to the volar locking plate group was used for analyses in this substudy of the PQ muscle (Figure 2).

Surgical technique

**Figure 3:**
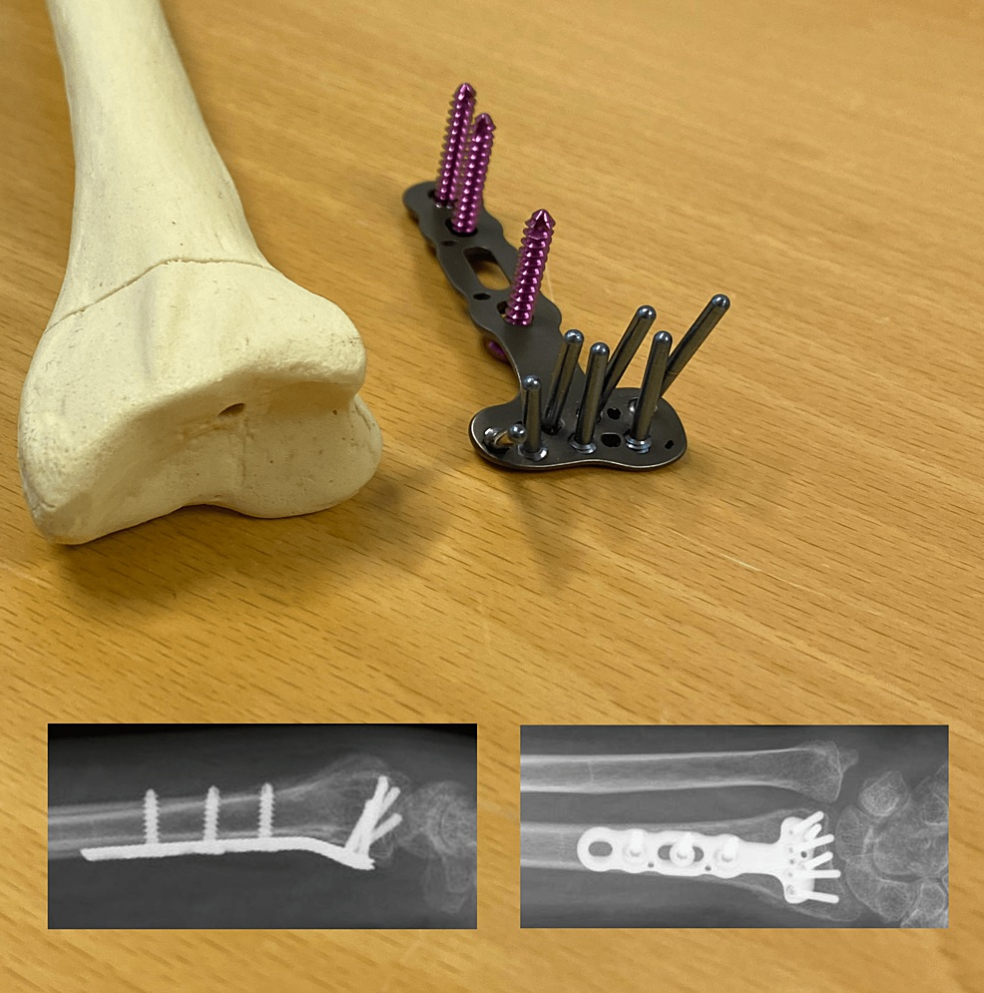
Radiographs and photograph showing a volar locking plate

**Figure 4:**
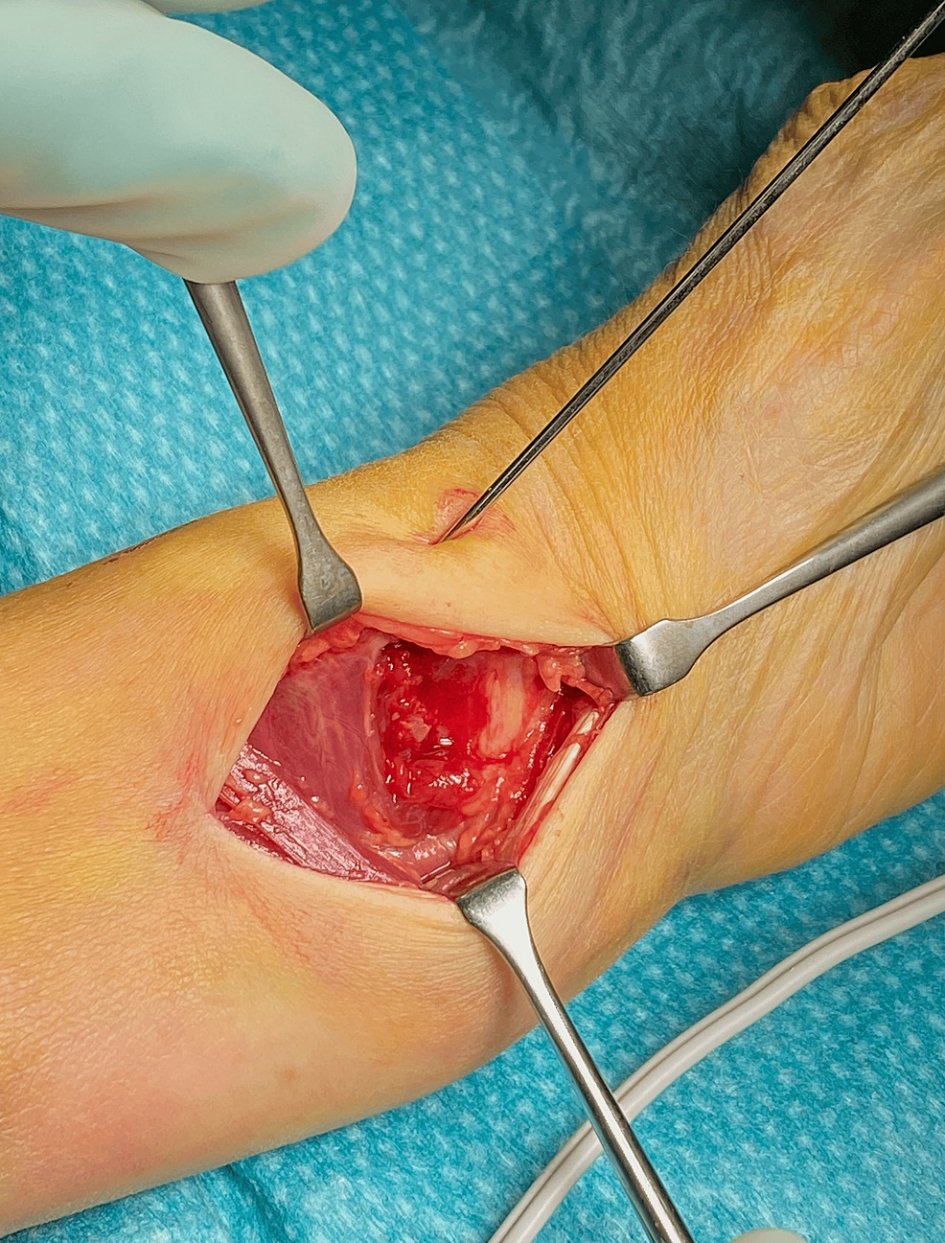
Surgical approach Intraoperative picture of ruptured pronator quadratus muscle prior to surgical release.

Surgery was performed according to the manufacturer’s guidelines. The DVR® (Depuy Synthes, Raynham, MA) is a volar locking plate with three or four cortical screws in the radial shaft, and five to nine smaller locking pegs or screws in the distal fragment of the radius (Figure 3). We used a standard volar approach [6] (Figure 4). The carpal tunnel was not routinely decompressed. Reduction and plate fixation were conducted under fluoroscopic surveillance. When possible, the PQ was sutured back to cover the distal part of the plate before skin closure.

Perioperative care

Surgery was mainly performed under general anesthesia. Cephalothin 2 g was administered intravenously 15-30 minutes preoperatively. Patients received a wound dressing and a wrist orthosis postoperatively. The orthosis was removed after two weeks but could be used intermittently during this period if desired. After six to eight weeks, the participants were instructed in basic exercises and received written illustrated instructions. The implants were not routinely removed.

Outcomes

Patients were assessed in the outpatient clinic at two weeks, six weeks, three months, six months, and one year, unblinded by a trained study nurse and a physiotherapist, who were not involved in enrollment or perioperative treatment. The primary outcome in the original RCT was the simplified version of the Disabilities of the Arm, Shoulder, and Hand Outcome Measure (QuickDASH) [7-9], which measures upper-limb function and has a score range of 0, representing excellent results, to 100, representing the worst possible results. The QuickDASH has a proposed minimal clinically important difference (MCID) of 14 (8-20) [10]. Secondary outcomes were the PRWE score, which is designed to measure wrist pain and disability subjectively during activities of daily living, with a score ranging from 0, representing excellent results, to 100, representing the worst possible results [11-13]. The PRWE score has a proposed MCID of 11.5 [14]. Health-related quality of life was also measured, using the three-level version of the EuroQol Group 5-Dimension Self-Report Questionnaire (EQ-5D), which includes both the EQ-5D index, which ranges from minus 0.59 (worst) to 1.00 (best) and the score on the EQ-5D visual analog scale (VAS), which ranges from 0 (worst) to 100 (best) [15]. Grip strength was measured in kilograms using a handheld dynamometer (Hydraulic Hand Dynamometer, MSD Europe, Belgium). Participants performed three maximum attempts for each measurement, and the average value was recorded. The MCID was set to 19.5% [16]. We did not adjust for hand dominance [17]. The ROM was measured with a goniometer. Patients underwent clinical examination for the detection of complications.

Statistical methods

We used the two-tailed Fisher’s exact tests for dichotomous variables and t-tests for numerical variables. Baseline characteristics were analyzed by normality tests. The nonparametric Mann-Whitney U test was used when there was a significant difference in variance.

## Results

Information on the integrity of the PQ was missing for five patients, leaving 55 patients for analysis. The median age was 67 years (55 to 88), and the one-year follow-up was 98%. In 28 patients, the PQ was intact, and in 27 patients, it was ruptured. In the intact group, 25 patients underwent repair of the PQ and three did not. Information was missing in one patient. In the ruptured group, 16 were repaired and 11 were not. Baseline characteristics were similar between the two groups (Table 1).

**Table 1:** Baseline and demographic characteristics. Baseline and demographic characteristics of patients according to the status of pronator quadratus at the injury. Values are expressed as mean, with standard deviation in parentheses, or as number of patients, with percentage in parentheses.

	Pronator quadratus not intact (n=27)	Pronator quadratus intact (n=28)	p-value
Age at fracture (years)	67.8 (8.1)	65.7 (7.3)	0.327
Women	25 (93)	23 (82)	0.422
Duration of surgery (minutes)	66.3 (19.6)	71.8 (16.4)	0.265
Injury of the dominant hand	10 (40)	15 (55.6)	0.283
Pain 6 hours postoperatively (Numerical Rating Scale 0-10)	2.1 (2.2)	2.3 (2.0)	0.656
Pain at discharge (Numerical Rating Scale 0-10)	2.1 (1.2)	2.0 (1.4)	0.699
Time from surgery to discharge (hours)	18.9 (10.4)	20.2 (9.0)	0.646

At one year, patients with an intact PQ had better QuickDASH scores (2.5 vs. 8.0, mean difference 5.5, 95% CI: 1.3 to 9.8, p=0.028). Patients with an intact PQ also had better EQ-5D Index scores after one year (0.94 vs. 0.85, mean difference 0.089, 95% CI: 0.004 to 0.174, p=0.031) (Table 2).

**Table 2:** Functional outcomes in all 55 patients according to status of pronator quadratus prior to surgery, regardless of repair or not. Number (n) varies because some information was missing for some patients. Values are expressed as mean, with standard deviation in parentheses, or as number of patients, with percentage in parentheses.

	Pronator quadratus not intact (n=27)	Pronator quadratus intact (n=28)	Mean difference (95% CI)	p-value
Mean (SD) Quick-DASH score				
At 2 weeks	43.8 (17.8) (n=27)	40.1 (16.9) (n=28)	3.8 (-5.4 to 12.9)	0.469
At 6 weeks	30.0 (17.8) (n=27)	25.3 (16.5) (n=28)	4.7 (-4.6 to 14.0)	0.363
At 3 months	16.9 (13.4) (n=26)	10.6 (9.2) (n=27)	6.4 (-0.03 to 12.8)	0.129
At 6 months	9.9 (10.2) (n=25)	5.5 (7.2) (n=27)	4.4 (-0.54 to 9.4)	0.087
At 1 year	8.0 (9.9) (n=26)	2.5 (3.9) (n=27)	5.5 (1.3 to 9.8)	0.028
Mean (SD) PRWE score				
At 2 weeks	41.0 (11.7) (n=27)	36.8 (11.3) (n=28)	4.2 (-2.0 to 10.4)	0.175
At 6 weeks	18.2 (9.3) (n=27)	15.4 (10.6) (n=28)	2.9 (-2.5 to 8.3)	0.165
At 3 months	8.8 (8.6) (n=26)	6.4 (6.9) (n=27)	2.5 (-1.8 to 6.8)	0.322
At 6 months	4.0 (6.3) (n=25)	2.3 (5.4) (n=27)	1.7 (-1.6 to 5.0)	0.296
At 1 year	1.6 (2.9) (n=26)	0.3 (0.9) (n=27)	1.3 (0.1 to 2.5)	0.058
Mean (SD) Eq-5d index score				
At 2 weeks	0.66 (0.19) (n=27)	0.72 (0.17) (n=28)	0.062 (-0.036 to 0.161)	0.229
At 6 weeks	0.68 (0.25) (n=27)	0.78 (0.15) (n=28)	0.103 (-0.008 to 0.214)	0.077
At 3 months	0.84 (0.14) (n=26)	0.85 (0.20) (n=27)	0.007 (-0.089 to 0.103)	0.295
At 6 months	0.82 (0.23) (n=25)	0.90 (0.12) (n=27)	0.085 (-0.018 to 0.188)	0.198
At 1 year	0.85 (0.19) (n=26)	0.94 (0.10) (n=27)	0.089 (0.004 to 0.174)	0.031
Mean (SD) Eq-5d visual analog scale				
At 2 weeks	69 (19.3) (n=27)	79 (12.0) (n=28)	9.9 (1.1 to 18.7)	0.064
At 6 weeks	75 (18.4) (n=27)	83 (10.2) (n=28)	7.3 (-0.89 to 15.4)	0.205
At 3 months	82 (15.0) (n=26)	86 (10.0) (n=27)	4.0 (-3.1 to 11.1)	0.451
At 6 months	83 (16.8) (n=24)	90 (7.5) (n=27)	6.4 (-1.2 to 14.0)	0.503
At 1 year	82 (18.8) (n=26)	90 (9.7) (n=27)	8.6 (0.25 to 17.0)	0.155

**Figure 5:**
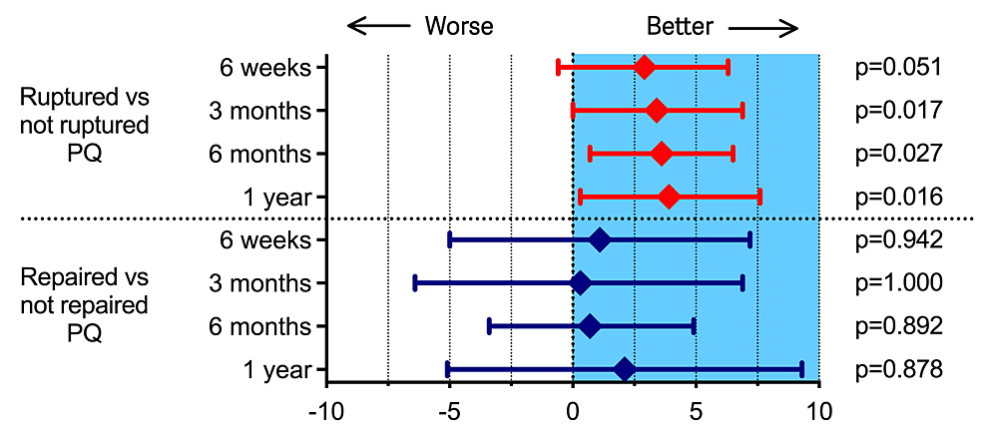
Grip strength: graphical results. Graph showing the mean difference in grip strength between ruptured vs not ruptured PQ (above) and between repaired vs not repaired PQ (below). The graph shows the superior grip strength in the not ruptured group at 3, 6, and 12 months, indicated by the entire 95% CI (shown by the whiskers) entirely above 0, in the blue area. It also shows no difference between the repaired and the not repaired group, indicated by the 95% CI crossing zero.

In patients with a ruptured PQ, we found that repair of the PQ did not improve grip strength or any other outcomes (Table 3). Patients with an identified intact PQ during surgery had better grip strength throughout the trial, after one year: 24 kg vs 20 kg (mean difference 3.9; 95% CI: 0.3 to 7.6, p=0.016) (Table 4, Figure 5). The intact PQ group regained 96% of the grip strength and the nonintact group regained 93%. There were no differences between any of the groups in terms of the PRWE score, EQ-5D VAS score, ROM, or VAS score up to one year (Table 5).

**Table 3:** Functional outcomes in 27 patients with injured pronator quadratus, according to repair or not during surgery. Number (n) varies because some information was missing for some patients. Values are expressed as mean, with standard deviation in parentheses, or as number of patients, with percentage in parentheses.

	Pronator quadratus not repaired (n=11)	Pronator quadratus repaired (n=16)	Mean difference (95% CI)	p-value
Mean (SD) Quick-DASH score				
At 2 weeks	41.7 (17.5) (n=11)	45.2 (17.0) (n=16)	3.5 (-10.4 to 17.4)	0.481
At 6 weeks	27.7 (15.2) (n=11)	31.6 (19.7) (n=16)	3.9 (-10.0 to 17.7)	0.512
At 3 months	16.9 (13.7) (n=11)	17.0 (13.7) (n=15)	0.05 (-11.3 to 11.4)	1.000
At 6 months	14.1 (12.7) (n=10)	7.1 (7.2) (n=15)	-7.0 (-16.6 to 2.6)	0.160
At 1 year	7.4 (9.9) (n=11)	8.5 (10.2) (n=15)	1.1 (-7.2 to 9.3)	0.683
Mean (SD) PRWE score				
At 2 weeks	36.9 (11.5) (n=11)	43.8 (11.3) (n=16)	6.9 (-2.2 to 16.1)	0.294
At 6 weeks	17.0 (9.0) (n=11)	19.1 (9.8) (n=16)	2.0 (-5.6 to 9.6)	0.481
At 3 months	8.5 (10.4) (n=11)	9.0 (7.3) (n=15)	0.53 (-7.2 to 8.3)	0.574
At 6 months	5.6 (9.2) (n=10)	2.9 (3.1) (n=15)	-2.6 (-9.3 to 4.1)	0.849
At 1 year	1.5 (3.2) (n=11)	1.7 (2.8) (n=15)	1.2 (-2.2 to 2.8)	0.507
Mean (SD) Eq-5d index score				
At 2 weeks	0.68 (0.17) (n=11)	0.64 (0.22) (n=16)	0.032 (-0.128 to 0.191)	0.790
At 6 weeks	0.77 (0.13) (n=11)	0.61 (0.29) (n=16)	0.152 (-0.187 to 0.323)	0.394
At 3 months	0.82 (0.15) (n=11)	0.86 (0.14) (n=15)	-0.047 (-0.169 to 0.075)	0.330
At 6 months	0.72 (0.31) (n=10)	0.88 (0.13) (n=15)	-0.157 (-0.386 to 0.071)	0.261
At 1 year	0.86 (0.12) (n=11)	0.84 (0.22) (n=15)	0.014 (-0.131 to 0.160)	0.610
Mean (SD) Eq-5d visual analog scale				
At 2 weeks	67 (23.2) (n=11)	71 (16.8) (n=16)	3.2 (-14.0 to 20.5)	0.680
At 6 weeks	75 (22.8) (n=11)	75 (15.6) (n=16)	0.17 (-16.5 to 16.9)	0.827
At 3 months	79 (18.8) (n=11)	84 (15.2) (n=15)	5.2 (-8.5 to 18.8)	0.721
At 6 months	81 (19.0) (n=9)	84 (15.2) (n=15)	3.6 (-13.0 to 20.2)	0.861
At 1 year	79 (20.1) (n=11)	83 (17.7) (n=15)	4.2 (-12.1 to 20.4)	0.799

**Table 4:** Wrist range of motion in degrees from neutral position, and grip strength in kg, in patients according to status of pronator quadratus prior to surgery, regardless of repair or not. Number (n) varies because some information was missing for some patients. Values are expressed as mean, with standard deviation in parentheses.

	Pronator quadratus not intact (n=27)	Pronator quadratus intact (n=28)	Mean difference (95% CI)	p-value
Mean (SD) dorsal range of motion (deg)				
At 2 weeks	32 (15) (n=27)	30 (14) (n=28)	2.0 (-5.8 to 9.9)	0.613
At 6 weeks	47 (15) (n=27)	45 (15) (n=28)	1.2 (-6.8 to 9.3)	0.866
At 3 months	57 (10) (n=26)	55 (11) (n=27)	1.9 (-3.8 to 7.6)	0.669
At 6 months	60 (10) (n=25)	61 (12) (n=27)	-1.2 (-7.2 to 4.8)	0.700
At 1 year	64 (9) (n=26)	64 (14) (n=27)	0.1 (-6.5 to 6.3)	0.728
Mean (SD) volar range of motion (deg)				
At 2 weeks	36 (11) (n=27)	37 (11) (n=28)	0.2 (-5.6 to 6.0)	0.966
At 6 weeks	46 (11) (n=27)	47 (12) (n=28)	0.3 (-5.9 to 6.6)	0.755
At 3 months	49 (9) (n=26)	53 (11) (n=27)	3.7 (-2.0 to 9.5)	0.222
At 6 months	55 (10) (n=25)	57 (11) (n=27)	2.2 (-3.5 to 7.9)	0.326
At 1 year	56 (10) (n=26)	61 (15) (n=27)	4.7 (-2.2 to 11.5)	0.167
Mean (SD) ulnar range of motion (deg)				
At 2 weeks	23 (10) (n=27)	24 (8) (n=28)	0.5 (-4.3 to 5.4)	0.619
At 6 weeks	27 (11) (n=27)	25 (10) (n=28)	-2.3 (-8.0 to 3.3)	0.448
At 3 months	30 (10) (n=26)	30 (7) (n=27)	0.4 (-4.5 to 5.2)	0.782
At 6 months	29 (7) (n=25)	31 (11) (n=27)	1.9 (-3.3 to 7.1)	0.607
At 1 year	31 (8) (n=26)	33 (11) (n=27)	2.4 (-3.0 to 7.9)	0.521
Mean (SD) radial range of motion (deg)				
At 2 weeks	17 (7) (n=27)	16 (5) (n=28)	-1.0 (-4.4 to 2.4)	0.624
At 6 weeks	21 (7) (n=27)	22 (8) (n=28)	0.6 (-3.4 to 4.6)	0.933
At 3 months	22 (7) (n=26)	25 (9) (n=27)	2.7 (-1.9 to 7.2)	0.387
At 6 months	22 (8) (n=25)	25 (9) (n=27)	3.5 (-1.1 to 8.1)	0.062
At 1 year	26 (7) (n=26)	26 (9) (n=27)	-0.2 (-4.8 to 4.3)	0.574
Mean (SD) pronation range of motion (deg)				
At 2 weeks	79 (19) (n=27)	79 (12) (n=28)	0.2 (-8.5 to 8.9)	0.315
At 6 weeks	80 (18) (n=27)	83 (5) (n=28)	3.1 (-4.3 to 10.4)	0.347
At 3 months	86 (5) (n=26)	86 (4) (n=27)	0.2 (-2.2 to 2.5)	0.786
At 6 months	88 (3) (n=25)	86 (6) (n=27)	-1.3 (-3.8 to 1.2)	0.300
At 1 year	88 (3) (n=26)	86 (14) (n=27)	-2.3 (-8.0 to 3.4)	0.271
Mean (SD) supination range of motion (deg)				
At 2 weeks	49 (15) (n=27)	54 (20) (n=28)	4.6 (-4.9 to 14.2)	0.266
At 6 weeks	59 (16) (n=27)	61 (17) (n=28)	1.4 (-7.3 to 10.3)	0.601
At 3 months	66 (13) (n=26)	72 (11) (n=27)	6.3 (-0.3 to 12.9)	0.058
At 6 months	70 (11) (n=25)	73 (10) (n=27)	3.5 (-2.4 to 9.3)	0.271
At 1 year	71 (12) (n=26)	71 (13) (n=27)	-0.3 (-7.0 to 6.5)	0.748
Mean (SD) grip strength (kg)				
At 6 weeks	10 (6.6) (n=27)	13 (6.0) (n=27)	2.9 (-0.6 to 6.3)	0.051
At 3 months	15 (7.0) (n=26)	19 (5.6) (n=27)	3.4 (-0.1 to 6.9)	0.017
At 6 months	18 (4.3) (n=25)	23 (6.1) (n=27)	3.6 (0.6 to 6.5)	0.027
At 1 year	20 (7.3) (n=26)	24 (5.9) (n=27)	3.9 (0.3 to 7.6)	0.016
Mean (SD) grip strength in percent of uninjured side (%)				
At 6 weeks	48 (24) (n=27)	55 (27) (n=27)	7.4 (-6.6 to 21.4)	0.406
At 3 months	68 (21) (n=26)	74 (22) (n=27)	5.6 (-6.1 to 17.4)	0.313
At 6 months	83 (15) (n=25)	86 (18) (n=27)	3.5 (-5.8 to 12.9)	0.458
At 1 year	93 (17) (n=26)	96 (19) (n=27)	3.3 (-6.7 to 13.2)	0.581

**Table 5:** Wrist range of motion in degrees from neutral position, and grip strength in kg, in 27 patients with injured pronator quadratus, according to repair or not during surgery. Number (n) varies because some information was missing for some patients. Values are expressed as mean, with standard deviation in parentheses.

	Pronator quadratus not repaired (n=11)	Pronator quadratus repaired (n=16)	Mean difference (95% CI)	p-value
Mean (SD) dorsal range of motion (deg)				
At 2 weeks	28 (13) (n=11)	35 (17) (n=16)	7.2 (-4.5 to 18.9)	0.251
At 6 weeks	47 (13) (n=11)	46 (17) (n=16)	-0.4 (-12.5 to 11.6)	0.680
At 3 months	55 (9) (n=11)	58 (10) (n=15)	2.3 (-5.7 to 10.3)	0.540
At 6 months	55 (10) (n=10)	63 (9) (n=15)	7.9 (-0.3 to 16.0)	0.048
At 1 year	63 (12) (n=11)	65 (7) (n=15)	2.6 (-6.1 to 11.4)	0.610
Mean (SD) volar range of motion (deg)				
At 2 weeks	35 (10) (n=11)	38 (12) (n=16)	3.0 (-5.6 to 11.6)	0.544
At 6 weeks	46 (14) (n=11)	47 (10) (n=16)	0.5 (-9.5 to 10.5)	0.753
At 3 months	48 (10) (n=11)	51 (9) (n=15)	3.2 (-4.7 to 11.0)	0.540
At 6 months	50 (9) (n=10)	57 (10) (n=15)	7.0 (-0.8 to 14.8)	0.055
At 1 year	52 (12) (n=11)	59 (7) (n=15)	7.0 (-1.3 to 15.3)	0.077
Mean (SD) ulnar range of motion (deg)				
At 2 weeks	22 (10) (n=11)	24 (10) (n=16)	1.6 (-6.6 to 9.9)	0.481
At 6 weeks	28 (13) (n=11)	27 (9) (n=16)	-0.8 (-10.6 to 9.0)	0.790
At 3 months	29 (13) (n=11)	30 (8) (n=15)	0.7 (-8.7 to 10.1)	0.574
At 6 months	27 (9) (n=10)	31 (6) (n=15)	3.9 (-2.9 to 10.7)	0.216
At 1 year	30 (9) (n=11)	32 (8) (n=15)	2.1 (-4.7 to 9.0)	0.540
Mean (SD) radial range of motion (deg)				
At 2 weeks	16 (7) (n=11)	18 (58 (n=16)	1.7 (-4.1 to 7.5)	0.865
At 6 weeks	21 (10) (n=11)	21 (5) (n=16)	0.4 (-6.5 to 7.3)	0.342
At 3 months	23 (9) (n=11)	21 (7) (n=15)	-2.0 (-8.6 to 4.5)	0.574
At 6 months	20 (9) (n=10)	24 (7) (n=15)	4.2 (-3.0 to 11.3)	0.091
At 1 year	25 (8) (n=11)	26 (6) (n=15)	1.7 (-4.5 to 7.9)	0.610
Mean (SD) pronation range of motion (deg)				
At 2 weeks	81 (14) (n=11)	77 (22) (n=16)	-3.5 (-17.8 to 10.7)	0.680
At 6 weeks	82 (11) (n=11)	79 (22) (n=16)	-2.7 (-16.0 to 10.6)	0.827
At 3 months	84 (6) (n=11)	87 (4) (n=15)	2.8 (-1.3 to 6.9)	0.180
At 6 months	87 (4) (n=10)	88 (3) (n=15)	1.5 (-1.6 to 4.6)	0.495
At 1 year	88 (2) (n=11)	89 (3) (n=15)	0.5 (-1.5 to 2.5)	0.357
Mean (SD) supination range of motion (deg)				
At 2 weeks	48 (17) (n=11)	50 (15) (n=16)	1.8 (-11.4 to 14.9)	0.645
At 6 weeks	60 (10) (n=11)	59 (19) (n=16)	-1.0 (-12.5 to 10.5)	0.790
At 3 months	70 (13) (n=11)	64 (13) (n=15)	-5.9 (-16.5 to 4.8)	0.540
At 6 months	68 (9) (n=10)	71 (12) (n=15)	2.5 (-6.3 to 11.3)	0.495
At 1 year	69 (10) (n=11)	72 (13) (n=15)	3.3 (-6.0 to 12.7)	0.259
Mean (SD) grip strength (kg)				
At 6 weeks	11 (8.5) (n=11)	10 (5.0) (n=16)	1.1 (-5.0 to 7.2)	0.942
At 3 months	16 (9.4) (n=11)	15 (4.9) (n=15)	0.3 (-6.4 to 6.9)	1.000
At 6 months	19 (5.4) (n=10)	19 (3.6) (n=15)	0.7 (-3.4 to 4.9)	0.892
At 1 year	22 (10.5) (n=11)	20 (3.7) (n=15)	2.1 (-5.1 to 9.3)	0.878
Mean (SD) grip strength in percent of uninjured side (%)				
At 6 weeks	49 (27) (n=11)	47 (23) (n=16)	-1.9 (-22.5 to 18.7)	0.942
At 3 months	66 (25) (n=11)	70 (18) (n=15)	3.6 (-15.1 to 22.3)	0.760
At 6 months	85 (19) (n=10)	82 (13) (n=15)	-3.2 (-17.7 to 11.3)	0.531
At 1 year	86 (19) (n=11)	98 (14) (n=15)	12.5 (-2.0 to 27.0)	0.164

There were no differences in reoperation or complication rates. Two patients in the intact group and one in the ruptured PQ group were later diagnosed with CTS and subsequently underwent carpal tunnel release. The PQ was repaired in the two patients in the intact group with CTS, but not in the patient with a ruptured PQ. One of the patients in the intact group with CTS had the plate removed during carpal tunnel release after eight months. One patient in the ruptured group had the plate removed after two months because of dorsal pain caused by a long protruding screw. Symptoms resolved in both patients. There were no cases of infection, tendon rupture, or delayed union.

## Discussion

Our study indicated that traumatic intramuscular rupture in the superficial head of the PQ after DRF may affect outcomes after DRF surgery via a volar approach. There was intramuscular rupture of the PQ in 27 patients (49%), and the PQ was intact in 28 (51%) patients. This finding supports our alternative hypothesis that the rupture of the PQ muscle, in addition to the L-shaped incision from surgery, which increases the risk of muscle denervation and scarring, can affect functional outcomes.

The literature on the integrity of the PQ muscle in DRF surgery is scarce. In their demographic data, Sonntag et al. reported 75% intact PQ muscles prior to DRF surgery, while in 16.7% of 72 patients, PQ muscles were not intact, but further analysis was not conducted on this matter [18]. In our study, we found an intact PQ muscle in 51% of the patients, demonstrating the importance of reporting this variable when discussing whether the PQ should be repaired after volar plating.

Our results are comparable to those of Swigart et al. who assessed PQ repair integrity following volar plate fixation for DRFs [19]. In their three-month follow-up study of 24 patients, they noted an intact PQ in 46% of patients. They reported no significant difference in ROM or grip strength between the intact and non-intact groups. In their study, patients with a less severe PQ injury tended to have better grip strength, but the difference was not statistically significant. In our study, we did find a statistically significant difference in grip strength in favor of the intact group.

An anatomical study conducted by Sakamoto et al. underlines important information on the innervation patterns of the anterior interosseous nerve to each head of the PQ muscle [20]. The branches innervating the superficial head run on the radius in a medial to lateral direction, and they advocate surgeries for DRFs to be conducted on the more lateral forearm. We speculate that laceration and fraying of the PQ muscle from the initial trauma, prior to surgical distal and radial detachment, may also potentially cause denervation of the muscle, thus affecting the outcome.

PQ muscle function after DRF may depend on several factors, including initial trauma, positioning and type of volar plate, muscle healing, retraction of an unrepaired muscle, and durability of the repair in a repaired muscle. Fang et al. found that the PQ muscle was replaced by scar tissue with nonintact muscle fibers in 103 patients upon plate removal [2]. PQ muscle fibers were observed in 23 patients, although the muscle fibers were loose, thin, and decreased in number. Additionally, the remaining muscle fibers exhibited varying degrees of adhesion to the volar plates, radial carpal flexor muscles, and interosseous membrane. They found no difference in outcome between patients with healed PQ muscle and those without healed PQ muscle. In contrast to our study, Fang et al. only included patients with an intact PQ muscle before release, indicating that scarring and fibrosis are not reserved for a ruptured PQ prior to surgery.

Some authors claim that patients experience an early effect of PQ repair. In a retrospective study of 63 patients, Pathak et al. reported improved pain relief and ROM at four weeks, and improved grip strength at three months in the repair group [21]. We could not confirm these early findings in our study. Others speculate that a too-tight repair can compromise PQ function. Hershman et al. conducted a prospective study of 112 patients whose forearm ROM was the primary outcome and reported poorer radial wrist deviation in the PQ repair group [22]. This finding contrasts with our findings, as we found no differences in ROM.

In a meta-analysis including five RCTs and six retrospective case-control studies, Lu et al. did not find any functional benefit of PQ repair after volar plate fixation of DRFs [23]. A subgroup analysis, however, revealed different effects on pronation strength in patients with different AO fracture types. Type B DRFs favored PQ repair and non-AO type B fractures favored no PQ repair. Only one study in the review provided grip strength data in comparison with the uninjured side, while the other studies only provided actual measured values in kilograms. In our study, we found lower mean grip strength in the group with a severed PQ muscle. The difference was 17%, which is lower than the proposed minimal clinically important difference (MCID) of 19.5%. This indicates that patients who present with a ruptured PQ after DRF have weaker grip strength. However, when individual results were compared to the opposite wrist, the two groups in our study recovered the same. We, therefore, suggest that grip strength data should be provided in comparison with the uninjured side when using grip strength as an outcome in treatment group comparisons. Other recent reviews and meta-analyses have concluded that PQ repair does not improve patient-reported outcome measures (PROMs), ROM, or grip strength [1,24,25], which is in line with our findings.

Finally, the protective effect of PQ repair on flexor tendon irritation, flexor tendon rupture, or symptomatic implant removal is not obvious, as adhesions and flexor pollicis longus rupture still occur after repair [2,26].

Our findings suggest that patients with a ruptured PQ after DRF who undergo additional surgical release of the muscle via volar plating, have lower QuickDASH scores and EQ-5D index scores after one year than patients with an intact PQ prior to surgical release. However, the difference in the QuickDASH may be of minor clinical relevance as it is lower than the proposed MCID. Our findings may indicate that PQ rupture might be a confounding factor in studies comparing PQ repair or not.

The present study has several limitations. We did not assess the integrity of the PQ muscle using ultrasound or MRI throughout the healing period. Thus, we are not able to describe the failure rate of the repair or retraction of the unrepaired muscles in relation to clinical outcomes. Next, the decision to repair the PQ was not randomized, and we did not perform pronation or supination strength testing.

The present study also has strengths. First, a high follow-up rate of 98%, and also the study population was quite homogenous, as patients with intraarticular fractures and younger patients were excluded from the original RCT.

## Conclusions

In this study, patients with an intramuscular rupture of the PQ muscle prior to volar plating in DRF surgery had an inferior clinical outcome compared to patients with an intact PQ. The differences were, however, less than those considered clinically relevant, and thus of uncertain clinical relevance to the individual patient. We encourage further larger randomized clinical trials to establish the clinical benefit of PQ repair and suggest that PQ integrity be reported. 
